# Genotype diversity of *Mycoplasma Hyopneumoniae* in Chinese swine herds based on multilocus sequence typing

**DOI:** 10.1186/s12917-021-03059-6

**Published:** 2021-11-08

**Authors:** Hui Zhang, Yuanyuan Wang, Lu Gao, Yan Wang, Rong Wei

**Affiliations:** China Animal Health and Epidemiology Center, No 369 Road, Qingdao, 266032 China

**Keywords:** *Mycoplasma hyopneumoniae*, MLST, Genotyping

## Abstract

**Background:**

Between 2018 and 2020, 989 clinical specimens from pigs showing clinical signs of a variety of swine diseases in 27 provinces in China were sampled and submitted for further testing. Nested PCR targeting the 16S rRNA gene of *Mycoplasma hyopneumoniae* and subsequent sequencing were used to analyse these specimens. *Mycoplasma hyopneumoniae*-positive samples were assayed by multilocus sequence typing (MLST). The aim of the study was to reveal the distribution of *M. hyopneumoniae* and determine the genotypes of *M. hyopneumoniae* in pig herds in China based on MLST.

**Results:**

Among these 989 samples, 199 samples were *M. hyopneumoniae*-positive. The *M. hyopneumoniae* positivity rate was 7.2% (35/494) in 2018, 18.4% (38/207) in 2019, and 43.8% (126/288) in 2020. In total, 47 samples were successfully assayed by MLST. Sixteen new *M. hyopneumoniae* sequence types from 9 provinces were recorded in the present study.

**Conclusions:**

This is the first report on sample positivity rates and molecular typing results for *M. hyopneumoniae* in swine herds in China. MLST has revealed high genotype diversity among *M. hyopneumoniae* from different provinces of China.

## Introduction

Enzootic pneumonia (EP) in pigs caused by *Mycoplasma hyopneumoniae* results in large economic losses worldwide. Isolation and cultivation of *M. hyopneumoniae* is complicated and time-consuming. Genotyping analysis of clinical materials rather than isolates of *M. hyopneumoniae* has been very useful in field investigations. Multilocus sequence typing (MLST) [1, 2], a molecular tool used in epidemiological investigations, has proven useful in investigations of outbreaks of EP in pigs and can be performed in clinical materials [3–5]. The data obtained can be easily shared among laboratories through online databases. While most MLST schemes utilize seven genes, it has been previously demonstrated that the use of only three genes (*adk*, *rpoB* and *tpiA*) provides the same resolution as the seven-gene scheme [1]. Molecular epidemiological studies on *M. hyopneumoniae* infection of pigs in China are rare, thus the aim of the present study was to reveal the distribution of *M. hyopneumoniae* and determine the genotypes of *M. hyopneumoniae* in pig herds in China based on MLST.

## Materials and methods

### Samples

**Fig. 1:**
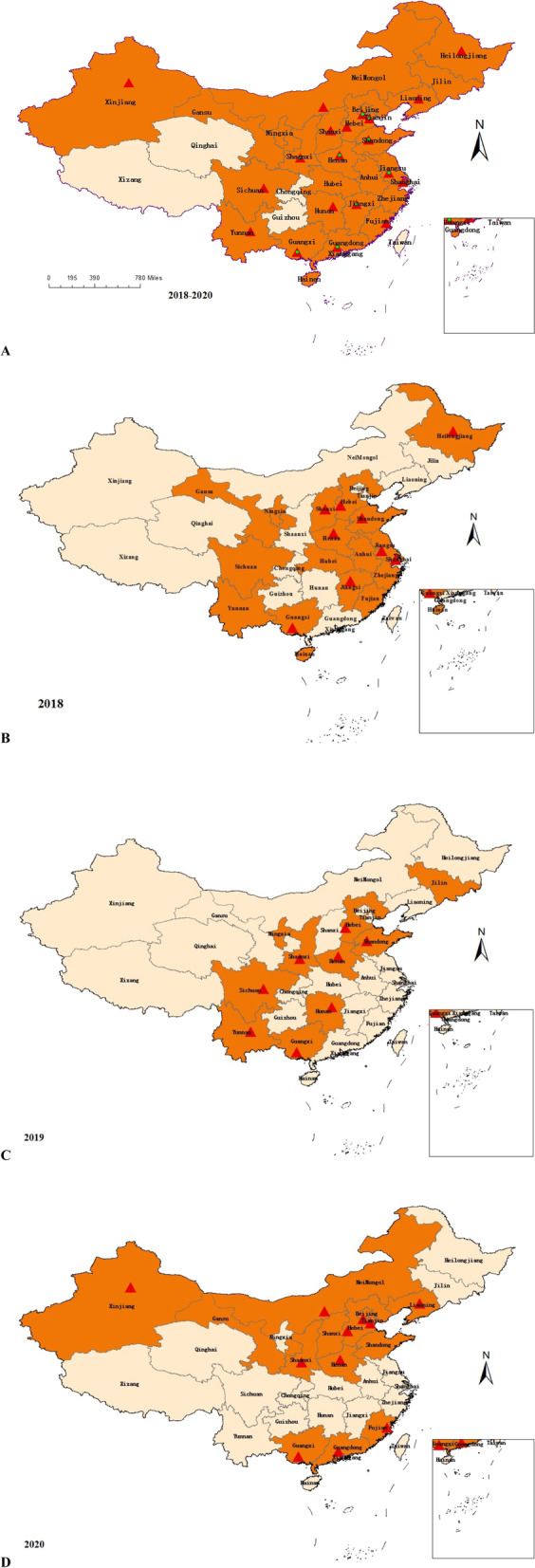
Distribution of samples. Samples were collected from herds in provinces coloured orange. Red triangles represent positive samples detected in the provinces. **A** Distribution of samples between 2018 and 2020. Green circles represent the province locations of the 26 positive herds. **B** Distribution of samples in 2018. **C** Distribution of samples in 2019. **D** Distribution of samples in 2020

Between 2018 and 2020, a total of 989 clinical specimens (homogenates derived from the lungs, spleen, liver, and lymph nodes) from 989 pigs with clinical symptoms of a variety of swine diseases in 215 pig herds in 27 provinces in China were sampled. Each homogenate sample was derived from one pig. Among them, 494 clinical specimens were sampled from 146 herds in 18 provinces in 2018 (Fig. 1B), 207 clinical specimens were sampled from 24 herds in 10 provinces in 2019 (Fig. 1C), and 288 clinical specimens were sampled from 45 herds in 14 provinces in 2020 (Fig. 1D), The sampled provinces from 2018 and 2020 are shown in Fig. 1A.

### DNA extraction, PCR amplification and sequencing

*M. hyopneumoniae* DNA was isolated from the homogenates using a magnetic particle-based sample preparation kit (Xi’an Tianlong Science & Technology Co., Ltd., Xi’an Shaanxi Province, China) in accordance with the manufacturer’s protocol. Nested-PCR [6] was used for the detection of *M. hyopneumoniae*. The first PCR amplified a 649-bp region of the 16S rRNA gene. After the second PCR, DNA segments measuring 352 bp in length were cloned into a pMD18-T vector (TaKaRa, Dalian Laoning Province, China) and sequenced by the BGI Group (Qingdao, China). Positive samples were confirmed by using the BLAST (http://blast.ncbi.nlm.nih.gov) and subjected to MLST.

### MLST

Nucleotide sequence data of the *adk*, *ropB*, and *tpiA* genes were obtained from *M. hyopneumoniae*-positive samples by Sanger sequencing. In brief, the three genes were amplified by primers and conditions described previously [1]. The PCR products were analysed by 1.5% agarose gel electrophoresis and purified with the Wizard® SV Gel and PCR Clean-Up System (Promega Corporation, Madison, WI, USA). DNA segments were sequenced by the BGI Group (Qingdao, China). Sequence data were submitted to the PubMLST database (http://pubmlst.org/mhyopneumoniae) for sequence type (ST) designation. The PubMLST database contains nomenclature-allele definitions that provide an identifier for every unique allele sequence and MLST profiles that index each unique combination of alleles with a ST. Sequences were aligned using the CLUSTAL W and cluster analysis was performed using the neighbour-joining method (Kimura two-parameter method) with Molecular Evolutionary Genetics Analysis (MEGA) software (version 6.0; https://www.megasoftware.net/). Bootstrap values were based on 1000 replicates. Values of Simpson’s index of diversity were calculated with the help of the Comparing Partitions website (http://www.comparingpartitions.info/index.php?link=Tool).

## Results and discussion

The results showed that 199 of 989 samples were *M. hyopneumoniae*-positive according to nested PCR and BLAST comparison,. The individual positivity rates for *M. hyopneumoniae* were 7.2% (35/494) in 2018, 18.4% (38/207) in 2019, and 43.8% (126/288) in 2020. The herd positivity rates for *M. hyopneumoniae* was 14.4% (21/146) in 2018, 54.2% (13/24) in 2019, and 55.6% (25/45) in 2020. The reason for the large percentage increase in *M. hyopneumoniae* detection may be that much attention had been paid to prevention and control of African Swine Fever (ASF) since its first outbreak in China in 2018(http://www.oie.int/). Also, vaccination against *M. hyopneumoniae* is not compulsory and vaccines are not widely used on farms. Another reason may be that a series of policies that reduced and eventually forbid the supplementation of antibiotics in animal feed were announced by the Minstry of Agriculture and Rural Affairs in march,2019.

Among the 199 samples, only 21 samples were sent with accompanying clinical sign information. These clinical signs included respiratory signs (dyspnoea, hyperpnea, coughing, fever or high fever), reproductive signs (abortion, weak piglets, mummified foetuses, or dead foetuses), neurological signs (ataxia, circling, paresis, or tremors), and gastrointestinal signs (vomiting or diarrhea). We assumed that most infections with *M. hyopneumoniae* were complicated by coinfection with other pathogens.

From 2018 to 2020, samples from 59 herds distributed in 20 of 27 provinces were positive for *M. hyopneumoniae*. Positive samples were detected from pig herds distributed throughout different regions of China (Fig. 1). Samples positive for *M. hyopneumoniae* were distributed in 9 of 18 provinces in 2018, 8 of 10 provinces in 2019, and 11 of 14 provinces in 2020 (Fig. 1). Seventy-three positive samples of 307 total samples were distributed in Hebei, Henan, and Guangxi provinces from 2018 to 2020, and 13 positive samples of 40 total samples were distributed in Shaanxi province in 2019 and 2020. No positive samples were detected in Gansu, Ningxia, Anhui, Zhejiang, Hubei, Hainan, or Jilin provinces. In these provinces, few samples were collected and most of them were collected in 2018. More samples should be collected from herds in these provinces to confirm the *M. hyopneumoniae* infection situation.

Direct contact and aerosol transmission are the main routes of *M. hyopneumoniae* transmission. Long-distance airborne transmission of the pathogen can occur [7]. Treatment with antibiotics such as tetracycline, macrolides and lincosamides can reduce clinical symptoms and the bacteria load in an individual [8], but it does not clear the infection. In a previous study, *M. hyopneumoniae* persisted in alveolar macrophages, interstitial macophages and type I pneumocytes in the lungs after experimental infection [9]. Therefore, a history of infection with *M. hyopneumoniae* in a herd could present a risk for persistence of the pathogen and reinfection. In China, the first *M. hyopneumoniae* strain was isolated in 1973 [10]. *M. hyopneumoniae* has existed for at least nearly 50 years and has evolved highly diverse genotypes.

In this study, *M. hyopneumoniae* genotyping was performed for the first time in China. Three housekeeping genes (*adk*, *rpoB* and *tpiA*) were detected in 47 of 199 *M. hyopneumoniae*-positive samples distributed among 26 herds. MLST allowed the amplification and sequencing of some individual genes (1–2) among 7 targets. However, the successful sequencing of all 7 targets was not possible for the majority of the samples. It is attributed to the low amount of *M. hyopneumoniae* DNA or the presence of unknown PCR inhibitors in samples [4, 5]. In the rest of the positive samples, only some of the three housekeeping genes were amplified. Alleles and STs from the examined herds are summarized in Table 1. In the independent analysis of each gene, 2 new adk alleles (41, 47), 5 new rpoB alleles (59, 60, 63, 64, 67), and 7 new tpiA alleles (57,58,59,60,62,71,73) were identified. Based on the new alleles and new allele combinations, the samples clustered into 16 new STs originating from 26 herds in 9 provinces of China. Analysis of the Simpson’s index of STs (0.935) showed high discriminatory power and high diversity of *M. hyopneumoniae* in China. Among these, ST128 was the most frequent ST identifed, this ST was found in 6 herds (23.08%) in 4 provinces that were geographically unrelated (Table 1). Except for ST128 and ST149 (detected in herds in Henan and Guangxi provinces), the STs were unique to the herds in each province. Since all the samples represented new STs based on the PubMLST database, we hypothesize that they belonged to exclusive Chinese genotypes. However, the ST data we obtained are limited and should be further studied in the future.

**Table 1:** Summary of positive herds by date and genotyping result

Herd ID	Country and region of herd	Date	*adk*^a^	*rpoB*^a^	*tpiA*^a^	ST^b^
H1	China, Jiangxi	2018	16	15	29	145
H2	China, Jiangsu	2018	23	59	57	128
H3	China,Shaanxi	2020	47	59	71	174
H4	China,Shaanxi	2020	47	59	71	174
H5	China,Beijing	2020	16	15	73	175
H6	China,Shandong	2018	16	60	58	129
H7	China,Shandong	2018	6	18	58	130
H8	China,Shandong	2019	16	60	58	129
H9	China,Shandong	2019	16	60	58	129
H10	China,Tianjin	2020	16	67	59	172
H11	China,Tianjin	2020	47	67	15	173
H12	China,Guangdong	2020	23	60	57	128
H13	China,Henan	2019	23	59	57	128
H14	China,Henan	2019	23	15	62	149
H15	China,Henan	2020	16	1	59	169
H16	China,Guangxi	2019	23	15	62	149
H17	China,Guangxi	2019	16	59	57	146
H18	China,Guangxi	2019	23	59	57	128
H19	China,Guangxi	2019	16	15	59	147
H20	China,Guangxi	2019	41	63	59	150
H21	China,Guangxi	2019	16	59	57	146
H22	China,Guangxi	2019	40	15	60	151
H23	China,Guangxi	2019	23	59	57	128
H24	China,Guangxi	2019	23	59	57	128
H25	China,Guangxi	2019	16	23	57	148
H26	China,Guangxi	2020	16	64	59	152
Strain 168	China,Gansu	1979	16	15	34	61
BQ14	France		10	2	1	17
Strain J	UK		5	16	16	28
Strain 232	USA		11	15	15	27
Strain 7448	Brazil		13	17	19	15
Simpson’s index			0.702(CI: 0.582–0.821)	0.822(CI: 0.729–0.914)	0.834(CI: 0.741–0.927)	0.935(CI: 0.876–0.995)

^a^Represents the housekeeping genes and the numbers below represent the allele definitions (an identifier for every unique allele sequence);

^b^Represents the MLST profiles that index unique combinations of alleles

Some STs (ST128, ST149) were present in different herds in different provinces. Among these provinces, Jiangsu and Guangxi were the farthest apart, at 1600 km. The results imply that free movement of pigs is associated with a high risk for the transmision of *M. hyopneumoniae*. Since the first ASF case in China was reported on 3 August 2018, the Chinese government has strengthened measures to control animal movement. The implementation of strict movement measures can also improve EP control.

**Fig. 2:**
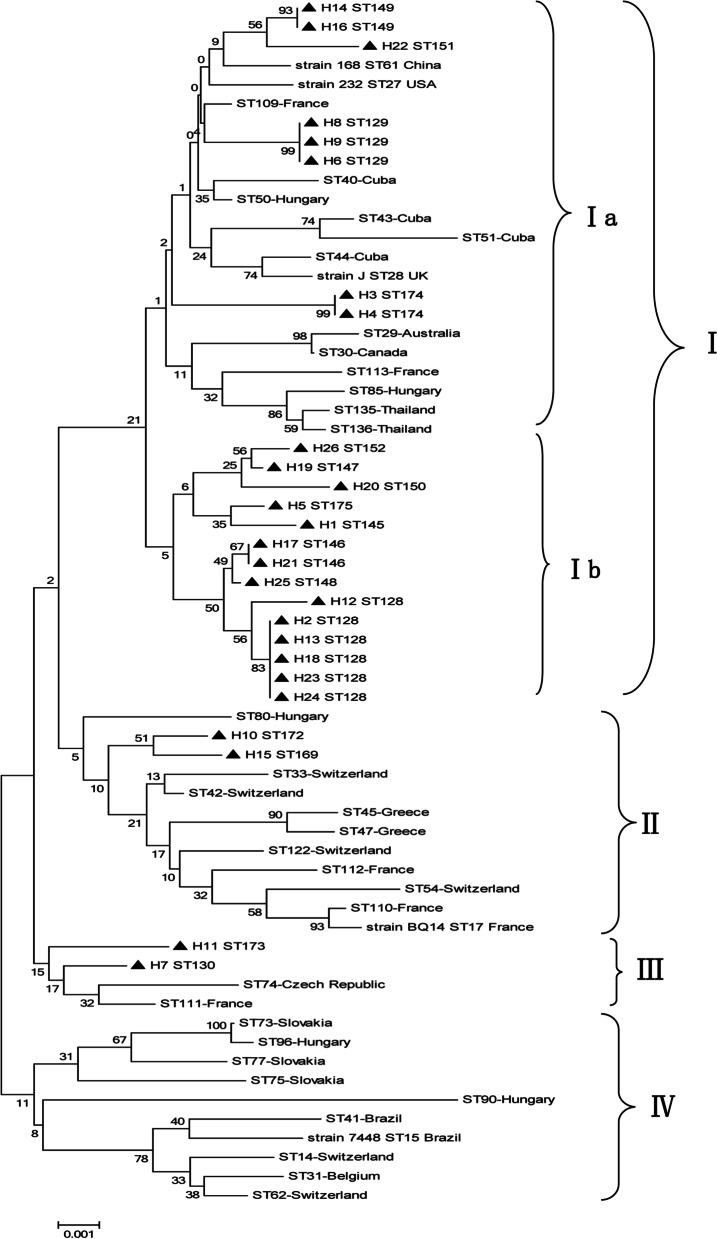
Phylogenetic tree of selected sequence types (STs) based on the *adk*, *rpoB* and *tpiA* sequences. The STs determined in this study are indicated by black triangles

Phylogenetic analysis showed that the 16 new STs were clustered into five clades with 37 STs from 15 countries: Ia, I b, II, III, and IV, as shown in Fig. 2. Ia and I b were the predominant clades prevalent in Chinese pig herds. Clade Ia included ST149, ST151, ST129, ST174 and STs from the USA, France, Cuba, Hungary, UK, Australia, Canada, and Thailand. Clade Ib contained all STs from China, which means *M. hyopneumoniae* has existed in China for a long time and formed individual cluster. Clade II included ST172, ST169 and STs from Hungary, Switzerland, Greece, and France. Subtype III included ST173, ST130 and STs from the Czech Republic and France. No Chinese STs were present in calde IV.

ST151 and ST149, which were detected in samples from different herds in Guangxi Province were phylogenetically related to ST61 representing Chinese strain 168, but only one of 3 alleles was the same (Fig. 2). This reference strain was isolated in 1979 from an EP outbreak in China [11]. This may imply that the original ST was replaced by the new STs via evolution.

ST152 and ST147, which were detected in samples from different herds from the same province (Guangxi), were phylogenetically close, with a 2-bp difference in *rpoB* gene (Fig. 2). ST129 was detected in samples from different herds in Shandong Province in 2018 and 2019 and formed a distinct cluster. ST174 detected in samples from Shaanxi Province, which is geographically isolated from the other provinces, also formed a distinct cluster. The STs of samples from these provinces are possibly prone to the formation of individual clusters.

ST146 and ST148 are phylogenetically close to ST128, which is the most common ST. ST175 and ST145 are phylogenetically close, differing in the *tpiA* gene by 4 bp. The three alleles of ST173 and ST130 are different but form a distinct cluster (Fig. 2). These STs are geographically unrelated but form a cluster, which implies the spread of *M. hyopneumoniae* among provinces.

## Conclusions

Positive samples were detected in pig herds distributed throughout different regions of China. MLST revealed high genotype diversity among *M. hyopneumoniae* isolates from different provinces of China. All the samples represented new STs based on the PubMLST database, and we hypothesize that they belong to exclusive Chinese genotypes. ST128, which was present in 4 provinces in different regions of China, was the most common ST in this study. The STs from most provinces are possibly prone to formation of their own cluster via evolution. Phylogenetic analysis showed that the Chinese STs belonged to four clades. It is difficult to elucidate the situation of *M. hyopneumoniae* in China in the long-term because of the high genotype diversity of the pathogen. This is the first study to report sample positivity rates and molecular typing results of *M. hyopneumoniae* in swine herds in China. To further understand the epidemiology and possible sources of *M. hyopneumoniae*, a surveillace plan for the disease should be formulated and implemented, and more samples could be collected and genotyped.

## Data Availability

The datasets generated and analysed during the current study are available in http://pubmlst.org/mhyopneumoniae.

## Abbreviations

EP: Enzootic pneumonia
MLST: Multilocus sequence typing
PCR: Polymerase chain reaction
ST: Sequence type
ASF: African swine fever
